# Diabetes and hypertension, as well as obesity and Alzheimer's disease, are linked to hypohydration-induced lower brain volume

**DOI:** 10.3389/fnagi.2014.00279

**Published:** 2014-10-13

**Authors:** Simon N. Thornton

**Affiliations:** Institut National de la Santé et de la Recherche Médicale, U_1116, Université de LorraineNancy, France

**Keywords:** dehydration, cognitive aging, angiotensin, aldsoterone, hypovolemia

In their excellent systematic review Meusel et al. (2014) put forward a hypothesis of a possible bias in imaging the brain during studies of cognitive aging with the inclusion of type 2 diabetics (T2DM) and hypertensive patients as part of the healthy controls. Their overview of metabolic and vascular changes associated with T2DM and hypertension raised again the interesting issue of what is similar between the two illnesses. It is known, furthermore, that a certain percentage of T2DM patients have also, or will go on to develop, hypertension. The same could be said for T2DM who have also, or who will develop, obesity and/or Alzheimer's disease. As these 4 diseases are currently affecting a large percentage of the population, especially the elderly, it might be of interest to consider what could be a common physiological parameter linking these illnesses that could affect also brain imaging and/or cognitive aging. A lower brain volume has been observed in Alzheimer's disease with or without mild cognitive impairment, in people with a high BMI (obesity), and in patients with progressive mild cognitive impairment (Ho et al., 2010; Liu et al., 2010; Raji et al., 2010). Furthermore, brain atrophy similar to that of preclinical Alzheimer's disease has been reported also in T2DM patients (Moran et al., 2013). These very interesting findings could suggest a common cause for altered brain function in these patients. It has been hypothesized that chronic hypovolemia (due to hypohydration) is perhaps one of the principal mechanisms behind the development of obesity, diabetes, hypertension, and even Alzheimer's disease (Fetissov and Thornton, 2009; Thornton and Benetos, 2011; Thornton, 2012). Now, if this were the case, then hypovolemia resulting from systemic dehydration could also reduce brain volume (Dickson et al., 2005; Duning et al., 2005). This “hypothesis” of dehydration is supported further by work showing that total body water decreases with age (Chumlea et al., 1999) as well as with increasing BMI (Ritz et al., 2008), thus suggesting that aged and/or obese and/or diabetic patients could be chronically dehydrated. Furthermore, the majority of medications used to treat cardiovascular disease block the renin-angiotensin (aldosterone) system yet this system is activated physiologically by hypovolemia (Thornton, 2010). Furthermore, high blood pressure levels have been associated with brain volume decreases (Beauchet et al., 2013), further adding support to overall dehydration–induced pathophysiology.

Dehydration has an important negative effect on cell metabolism (Schliess and Häussinger, 2003; Fetissov and Thornton, 2009; Thornton et al., 2009) which may go part way to explaining the association of cognitive dysfunction with decreases in brain volume and signs of atrophy. It has been suggested that increased drinking would go some way to alleviating some of the signs of the dehydration and the decreased metabolism. An increased fluid intake would also allow the brain to rehydrate (Duning et al., 2005). It would be very interesting to investigate just what influence these changes in brain volume, and thus neuronal volume and metabolism with dehydration and rehydration, would have on cognitive impairment associated with aging and associated with T2DM, obesity, as well as cardiovascular, and Alzheimer's disease.

In contrast to what has been found in the pathophysiology mentioned above, dehydration in healthy humans does not appear to have a great influence on brain volume changes (Kempton et al., 2009). Whereas in healthy adolescences, again with no obvious changes in brain volume, dehydration appeared to negatively impact “executive functions such as planning and visuo-spatial processing” where individuals needed to exert a higher level of neuronal activity in order to achieve the same performance level (Kempton et al., 2011). It should be noted that these effects were noticed with short-term dehydration, whereas the overall effects of long-term, or chronic, dehydration appear to be more global affecting various parts of the body (obesity, insulin resistance, blood pressure) as well as the brain (cognitive dysfunction). A screening method for whole body dehydration (angiotensin and aldosterone hormone levels) should perhaps be recommended as a new norm for imaging studies of cognitive aging.

## Conflict of interest statement

The author declares that the research was conducted in the absence of any commercial or financial relationships that could be construed as a potential conflict of interest.
